# By protecting against cutaneous inflammation, epidermal pigmentation provided an additional advantage for ancestral humans

**DOI:** 10.1111/eva.12858

**Published:** 2019-09-24

**Authors:** Tzu‐Kai Lin, Mao‐Qiang Man, Katrina Abuabara, Joan S. Wakefield, Hamm‐ming Sheu, Jui‐chen Tsai, Chih‐Hung Lee, Peter M. Elias

**Affiliations:** ^1^ Department of Dermatology Hualien Tzu Chi Hospital Buddhist Tzu Chi Medical Foundation Hualien Taiwan; ^2^ School of Medicine Tzu Chi University Hualien Taiwan; ^3^ Department of Dermatology VA Med Ctr/UCSF San Francisco California; ^4^ Program for Clinical Research Department of Dermatology UC San Francisco School of Medicine San Francisco California; ^5^ Department of Dermatology National Cheng Kung University College of Medicine Tainan Taiwan; ^6^ Institute of Clinical Pharmacy and Biopharmaceutical Sciences College of Medicine National Cheng Kung University Tainan Taiwan; ^7^ Department of Dermatology Kaohsiung Chang Gung Memorial Hospital and Chang Gung University College of Medicine Kaohsiung Taiwan

**Keywords:** barrier function, epidermis, evolution, inflammation, melanin, pH, pigmentation

## Abstract

Pigmentation evolved in ancestral humans to protect against toxic, ultraviolet B irradiation, but the question remains: “what is being protected?” Because humans with dark pigmentation display a suite of superior epidermal functions in comparison with their more lightly pigmented counterparts, we hypothesized and provided evidence that dark pigmentation evolved in Africa to support cutaneous function. Because our prior clinical studies also showed that a restoration of a competent barrier dampens cutaneous inflammation, we hypothesized that resistance to inflammation could have provided pigmented hominins with yet another, important evolutionary benefit. We addressed this issue here in two closely related strains of hairless mice, endowed with either moderate (Skh2/J) or absent (Skh1) pigmentation. In these models, we showed that (a) pigmented mice display a markedly reduced propensity to develop inflammation after challenges with either a topical irritant or allergen in comparison with their nonpigmented counterparts; (b) visible and histologic evidence of inflammation was paralleled by reduced levels of pro‐inflammatory cytokines (i.e., IL‐1α and INFα); (c) because depigmentation of Skh2/J mouse skin enhanced both visible inflammation and pro‐inflammatory cytokine levels after comparable pro‐inflammatory challenges, the reduced propensity to develop inflammation was directly linked to the presence of pigmentation; and (d) furthermore, in accordance with our prior work showing that pigment production endows benefits by reducing the surface pH of skin, acidification of albino (Skh1) mouse skin also protected against inflammation, and equalized cytokine levels to those found in pigmented skin. In summary, pigmentation yields a reduced propensity to develop inflammation, consistent with our hypothesis that dark pigmentation evolved in ancestral humans to provide a suite of barrier‐linked benefits that now include resistance to inflammation.

## 1 INTRODUCTION

It is generally acknowledged that two of the three unique features of human skin—widespread dispersion of sweat glands and shedding of their furry pélage—evolved to facilitate the dissipation of heat as ancestral *Homo* began to venture out onto open savannahs (rev. in Jablonski & Chaplin, 2017). The underlying epidermis of furred mammals is pale (with the exception of polar bears). Indeed, genetic studies have shown that the skin of ancestral humans only began to darken after *Homo erectus* shed his furry mantle over one million years ago. But the evolutionary “driver” of this third, unique feature of human skin—deep cutaneous pigmentation—is hotly debated. While there is wide agreement that dark pigmentation (DP) arose in response to the relentless assault of toxic ultraviolet (UV‐B) irradiation at sub‐Saharan latitudes (Ibid.), the identity of the threatened structural or biochemical target is disputed. A still‐prevalent explanation for the evolution of DP holds that it emerged to prevent the development of UV‐induced skin cancers (Brenner & Hearing, 2008; Lin & Fisher, 2007), a theory that has recently fallen out of favor, because: (a) neolithic humans remained dark after migrating into less UV‐B intense latitudes in south‐central Europe (Olalde et al., 2014); (b) despite the oft‐cited Loomis diagram (Loomis, 1967), the latitude‐dependent loss of pigmentation did not occur except in Europeans of the far North; (c) the great majority of nonmelanoma skin cancers emerge long after peak reproductive years (Elias & Williams, 2013; Jablonski & Chaplin, 2017); and few fossils of ancestral humans were aged over 40 years (cited in Elias & Williams, 2015).

The current, prevailing paradigm holds that DP evolved to protect against congenital anomalies attributable to UV‐induced destruction of the photosensitive vitamin, folic acid (Chaplin & Jablonski, 2009; Jablonski & Chaplin, 2010, 2017). While in vitro irradiation photodegrades folic acid and its active metabolites (Fukuwatari, Fujita, & Shibata, 2009; Jones, Lucock, Veysey, & Beckett, 2018; Juzeniene, Thu Tam, Iani, & Moan, 2009; Off et al., 2005; Steindal, Tam, Lu, Juzeniene, & Moan, 2008), as well as inhibiting enzymes that are involved in folate metabolism (Jones et al., 2018), a substantial body of evidence contradicts this putative relationship (rev in Elias & Williams, 2015, 2018). Not only is the overall prevalence of congenital neuroanomalies very low (<1:2,000 births), but the great majority of them were also not severe enough to have compromised reproductive fitness (Northrup & Volcik, 2000). Even more convincing are studies in patients receiving repeated, supraphysiologic doses of either incident UV‐B or UV‐A irradiation for the treatment of inflammatory skin disorders. With one exception (El‐Saie, Rabie, Kamel, Seddeik, & Elsaie, 2011), these studies have shown little or no decline in postirradiation levels of circulating folic acid (e.g., Cicarma et al., 2010; Gambichler, Bader, Sauermann, Altmeyer, & Hoffmann, 2001; Juzeniene, Stokke, Thune, & Moan, 2010). Moreover, though folic acid is sensitive to UV light in the “test tube” (Fukuwatari et al., 2009; Juzeniene, Thu Tam, Iani, & Moan, 2013), little incident UV‐B reaches the deeper layers of skin (Anderson & Parrish, 1981; Honigsmann, 2002), where this vitamin circulates. Finally, dietary sources of folic acid have always been both readily and widely available (Shane, 2010). Thus, it seems unlikely that protection against UV‐induced, folic acid degradation “drove” the evolution of dark pigmentation (Northrup & Volcik, 2000; Rasmussen et al., 1998; Rayburn, Stanley, & Garrett, 1996).

Our prior studies of DP versus lightly pigmented (LP) humans suggest a third possible “driver” of epidermal pigmentation. Though several epidemiologic studies report a paradoxically greater, rather than a lesser propensity to develop inflammatory dermatoses in DP humans, these disorders also tend to be more severe in DP humans (Abuabara et al., 2019; DeFelice & Yousef, 2008; Moore et al., 2004; Vachiramon, Tey, Thompson, & Yosipovitch, 2012; Wegienka et al., 2012; Williams et al., 1995). To address this apparent paradox, we assessed whether DP bestows yet another, barrier‐related advantage by increasing inflammatory thresholds, potentially protecting ancestral humans from a variety of potentially harmful, pro‐inflammatory insults. Yet, DP humans demonstrate both superior epidermal permeability barrier function and stratum corneum cohesion in comparison with LP subjects (Gunathilake et al., 2009; Reed, Ghadially, & Elias, 1995), independent of race, ethnicity, latitude of residence, gender, or occupation. Because “resetting” the surface pH of LP humans downward to levels found in DP subjects bestowed them with optimal cutaneous function (Gunathilake et al., 2009), the benefits of DP can be attributed to the reduced surface pH of DP individuals.

Our results in closely related mouse strains with divergent pigmentation show that pigmented mouse skin resists both irritant and allergic insults better than the skin of albino mice, and that this resistance can be attributed to both the presence of cutaneous pigmentation and the impact of the lower pH endowed by pigmentation (Man et al., 2014). Together, these results provide further support for the hypothesis that deep pigmentation evolved in ancestral humans (Elias, Menon, Wetzel, & Williams, 2009, 2010) in order to support epidermal structure and function (Elias & Williams, 2013).

Because a reduced skin surface pH offers many advantages for epidermal structure and function (rev. in Elias, 2017), we hypothesized that DP initially evolved to support cutaneous structure and function in the recently denuded skin of ancestral humans (Elias et al., 2009, 2010). As they ventured out onto open savannahs (Elias & Williams, 2013), toxic doses of UV‐B, which compromise the permeability barrier (Biniek, Levi, & Dauskardt, 2012; Haratake et al., 1997; Holleran et al., 1997), coupled with the extreme xeric stress of this region due to both epochal drying (e.g., Blome, Cohen, Tryon, Brooks, & Russell, 2012) and repeated megadroughts (e.g., DeMenocal, 2004) would have inevitably placed further stress on the epidermis of denuded skin. In this context, the evolution of DP would have provided important benefits: first and foremost, it would have “reset” the threshold for UV‐B‐induced toxicity, while simultaneously providing the additional, substantial benefits of “suberythemogenic” UV‐B. In contrast to the harmful impact of erythemogenic UV‐B, suberythemogenic doses enhance (a) permeability barrier function, minimizing losses of internal water into the desiccating milieu of sub‐Saharan Africa; and (b) innate immunity against microbial pathogens (Hong et al., 2008). Finally, in pertinent clinical studies, we showed that restoration of normal barrier function in inflamed human and murine skin provides an additional benefit—it reduces the severity of inflammation (rev in Elias, 2017; Elias, Wakefield, & Man, 2018). By comparing the propensity to develop inflammation in two genetically related mouse strains that differ in the extent of their pigmentation, we addressed the clinical paradox of more frequent severe inflammatory dermatoses in DP humans.

## 2 METHODS AND MATERIALS

### 2.1 Animals and materials

Female hairless, nonpigmented mice (Crl:Skh1‐ Hr^hr^/Hr^hr^), referred herein as Skh1 mice, were obtained from Charles River Laboratories, while pigmented (Skh2/J‐ Hr^hr^/Hr^hr^) hairless mice (Skh2/J) were from Jackson Laboratory. The latter mice display diffuse pigmentation (Man et al., 2014), which is further accentuated in the skin of their ears. All mice were fed a standard mouse diet (Ralston‐Purina Co), had access to tap water ad libitum, and were studied between 6 and 8 weeks of age. Mice were housed in a temperature‐ and humidity‐controlled, SPF facility. Affinity‐purified, rabbit anti‐mouse antibodies to TNFα, IL‐1α, and IL‐1β were purchased from Abcam. Methanol, ethanol, propylene glycol (PG), 4‐ethoxymethylene‐2‐phenyl‐2‐oxazolin‐5‐one (Ox), and 12‐O‐tetradecanoylphorbol 13‐acetate (TPA), hydroquinone (HQ), and lactobionic acid (LBA) were from Sigma. Fontana–Masson (melanin staining kit) was from Abcam. Samples for melanin staining were fixed in 10% buffered formalin, embedded in paraffin, and routinely processed. Five‐micrometer sections were used for the staining, using the Fontana–Masson method, following the protocol provided in the Fontana–Masson Kit (Abcam).

### 2.2 Experimental procedures

#### 2.2.1 Irritant contact dermatitis model

Initially, for depigmentation, 4% hydroquinone (HQ), dissolved in absolute ethanol, was applied twice daily onto the dorsal skin of depigmented Skh2/J mice for ten days. The phorbol ester, 12‐O‐tetradecanoylphorbol‐13‐acetate (TPA) was utilized as the irritant to induce Irritant contact dermatitis (ICD) in pigmented and nonpigmented skin. Female hairless mice (Skh1 and Skh2/J) were divided into five groups: (a) control SKH1; (b) TPA‐treated Skh1; (c) control Skh2/J; (d) TPA‐treated Skh2/J; and (e) TPA‐treated, depigmented Skh2/J groups. 0.001% TPA (in absolute ethanol) was applied once or twice daily to the dorsal skin for 10 days. Twenty‐four hours after the last challenge, transepidermal water loss (TEWL) and stratum corneum (SC) hydration were assessed with respective probes connected to a Tewameter (MPA5, Courage & Khazaka), as described previously (Hatano et al., 2011; Man et al., 2008). Skin samples for real‐time PCR (rtPCR) were taken immediately after the last functional determinations, as below (Man et al., 2008).

#### 2.2.2 Atopic dermatitis (AD) model

4‐Ethoxymethylene‐2‐phenyl‐2‐oxazolin‐5‐one (oxazolone, Ox) was utilized to induce AD in pigmented and nonpigmented mice, divided into the same five cohorts described above. Female hairless mice (Skh1 and Skh2/J) were first sensitized with 3% Ox (in absolute ethanol), applied topically to dorsal skin and flanks. After one week, 0.01% Ox was applied once daily to the dorsal and flank skin for 21 days, producing the AD model (Man et al., 2008).

### 2.3 Depigmentation/acidification in animal models

#### 2.3.1 Irritant contact dermatitis model

Female Skh2/J hairless mice were divided into the four cohorts: control Skh2/J, TPA‐treated Skh2/J, TPA‐treated, depigmented Skh2/J, and TPA‐treated depigmented/acidified Skh2/J group. 4% hydroquinone (HQ), dissolved in absolute ethanol, was applied twice daily onto the dorsal skin of depigmented Skh2/J mice for ten days. 0.005% TPA was then applied once daily to dorsal skin and flanks for up to 21 days. The length of TPA treatments again depended upon the appearance of visible inflammation in the Skh2/J mice. During the 21 days of TPA challenges, HQ was applied twice daily to the dorsal skin and flanks of depigmented Skh2/J mice, with the first application at least 1 hr after the prior daily TPA challenge. 10% lactobionic acid (LBA) (dissolved in PG:water:ethanol [2:2:6 vols]) was applied daily after HQ treatment of one depigmented group. 24 hr after the last challenge, skin surface pH, melanin indices and the extent of erythema were assessed with a pH900 pH probe and MEXAMETER^®^ 18 probe, respectively, connected to the MPA5, as above. Thereafter, skin samples were obtained for rtPCR (see below).

#### 2.3.2 Atopic dermatitis (AD) model

Skh2/J mice were divided into the same 4 groups as above. Female Skh2/J hairless mice were first sensitized with 3% Ox, applied topically to both dorsal and flank skin. 4% Hydroquinone (HQ) dissolved in absolute ethanol was applied twice daily to the dorsal skin and flanks of one of the depigmented SKH2/J group for the following week, following which, 0.01%~0.05% Ox was applied once daily to the dorsal skin and flanks for 17 days. The concentration of Ox applied depended upon the extent of visible dermatitis in Skh2/J mice with AD‐like dermatitis. During the 17 days of Ox challenges, hydroquinone (HQ) was applied twice daily, with the first application at least one hour after the prior Ox challenge. In one cohort, 10% lactobionic acid (LBA; dissolved in PG:water:ethanol [2:2:6 vols]) was applied daily after the prior HQ treatment and 24 hr after the last Ox challenge. Then, melanin and erythema indices, as well as skin surface pH, were assessed as above, and skin samples were obtained for rtPCR (see below).

### 2.4 Quantitative PCR for mRNA expression

Total epidermal RNA was isolated from sheets of hairless mouse epidermis (Proksch, Elias, & Feingold, 1991), using TRIzol Reagent (Sigma‐Aldrich; catalog number T9424), according to the manufacturer's directions. First‐strand cDNA was synthesized from 1 μg of total RNA with the Transcriptor First‐Strand cDNA Synthesis Kit (Roche, catalog number 04897030001) in a total volume of 20 μl. Real‐time PCR utilized 60 ng of reversed‐transcribed cDNA, 300 nm each of forward and reverse primers, and 10 μl of SYBR^®^ Green PCR Master Mix (Thermo Fisher Scientific) in a final volume of 20 μl in 96‐well plates using the new Step One(TM) Real‐Time PCR System. Quantitation was performed by the comparative threshold cycle method with mouse glyceraldehyde‐3‐phosphate dehydrogenase used for normalization. The primer sequences for tumor necrosis factor‐α (TNF‐α) were 5′‐ TTGAGATCCATGCCGTTG‐3′ (forward) and 5′‐CTGTAGCCCACGTCGTAGC‐3′ (reverse). The primer sequences for interleukin 1α (IL‐1α) were 5′‐ TGGGTATCTCAGGCATCTCC‐3′ (forward) and 5′‐ ATCAGTACCTCACGGCTGCT‐3′ (reverse). The primer sequences for interleukin 1β (IL‐1β) were 5′‐ TCTTCTTTGGGTATTGCTTGG‐3′ (forward) and 5′‐ TGTAATGAAAGACGGCACACC‐3′ (reverse). The primer sequences for mouse glyceraldehyde‐3‐phosphate dehydrogenase (GAPDH) were 5′‐ CTGCTTCACCACCTTCTTGA‐3′ (forward) and 5′‐ AAGGTCATCCCAGAGCTGAA‐3′ (reverse). For all PCR studies, all samples were run in triplicate, and expression of mRNAs was normalized to GAPDH mRNA, and the expression of mRNAs relative to mRNA levels in the epidermis of control mice was calculated and expressed as percent of controls (setting controls at 100%).

### 2.5 Statistical analyses

Data are expressed as the means ± *SEM*. Unpaired two‐tailed Student's *t* test with Welch's correction was used to determine significant differences when two groups were compared, and a one‐way ANOVA with a post‐Tukey test or Dunnett postcorrection was used to determine significant differences when three or more groups were compared. Further details about statistical methods can be found in the figure legends.

## 3 RESULTS

### 3.1 Less Severe ICD and AD in Pigmented than in Albino Mice

**Figure 1:**
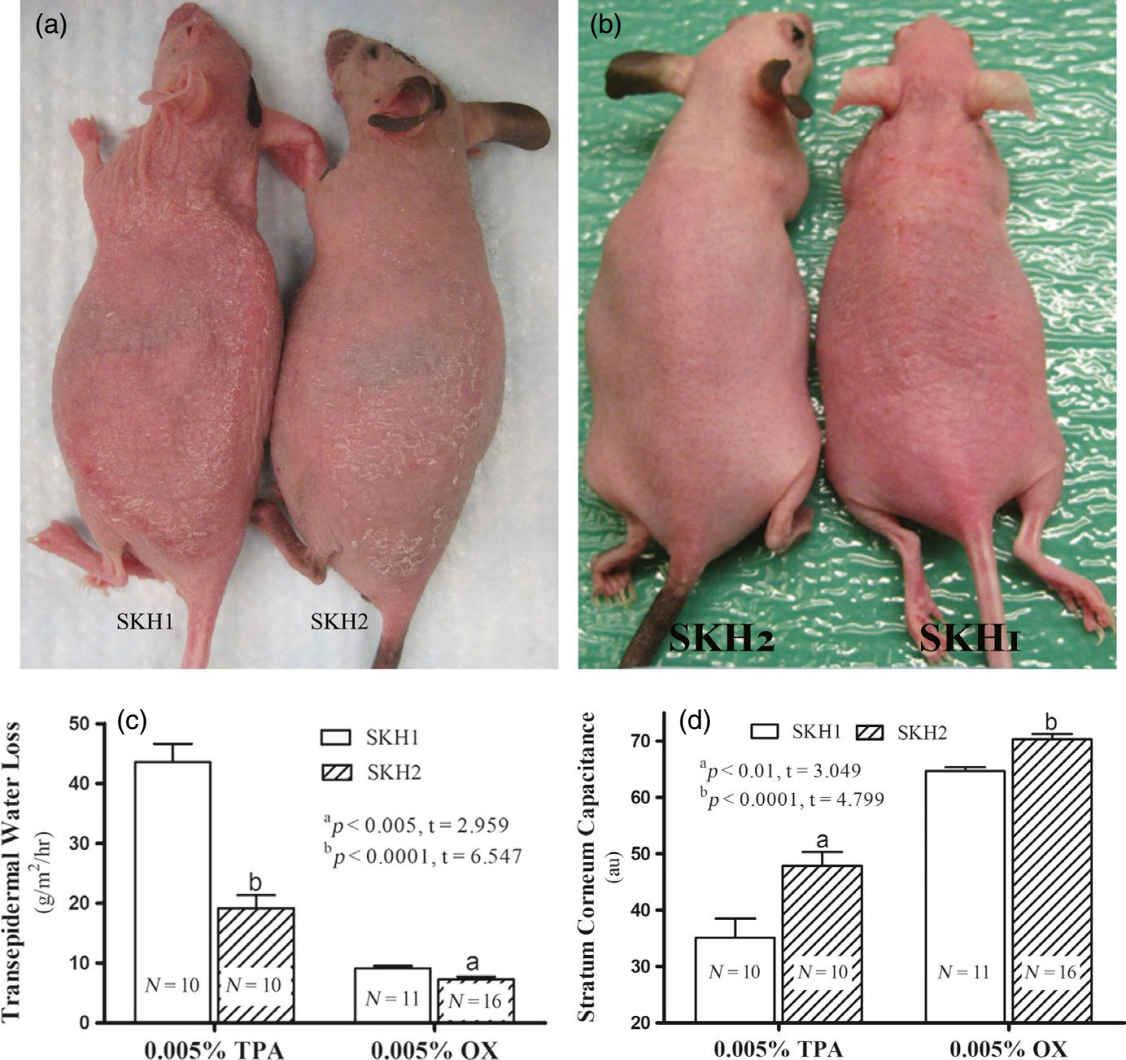
Pigmentation increases inflammatory thresholds. Irritant (ICD) and atopic dermatitis (AD)‐like inflammation were induced by repeated topical applications of TPA and oxazolone (Ox), respectively, as detailed in Methods. Figure 1a depicts mice treated with TPA, while Figure 1b shows mice treated repeatedly with Ox. Figure 1c & d presents changes in TEWL levels and stratum corneum hydration, respectively, in pigmented (Skh2/J) versus nonpigmented (Skh1) mice. Unpaired two‐tailed *t* test was used to determine the significance between the two groups. *p* values (Skh1 vs. Skh2 mice) are indicated as “a” and “b” in Figure 1c and d. *N* = 10 for mice treated with TPA; *N* = 11 for Skh1 mice treated with Ox; *N* = 16 for Skh2/J mice treated with Ox

Though cutaneous pigmentation appears most prominent in the ear skin of Skh2/J mice, our prior studies showed that the entire skin surface of Skh2/J epidermis is also uniformly populated by a mat of melanocytes, with melanin production evident histologically (Man et al., 2014). In contrast, the skin of Skh1 mice, though populated by melanoblasts, is completely devoid of functional melanocytes and melanin (Ibid.). Hence, we initially compared the severity of inflammatory responses on the flanks of pigmented (Skh2/J) versus nonpigmented (Skh1) hairless mice. Visible inflammation was markedly more severe in Skh1 than in Skh2/J mice after comparable challenges with either the phorbol ester, TPA (irritant contact dermatitis [ICD] model; Sheu et al., 2002), or the universal hapten, oxazolone (Ox), which produces an atopic dermatitis (AD)‐like phenotype in mice (Man et al., 2008; Figure 1a and b). Specifically, clinical features of inflammation (erythema and scaling) were much less apparent in both TPA‐treated and Ox‐challenged Skh2/J mice than in comparably challenged Skh1 mice. Finally, these differences in the extent of inflammation were paralleled by differences in epidermal function that persist, even in the aftermath of pro‐inflammatory challenges (Figure 1c and d).

### 3.2 More Severe ICD and AD Following Depigmentation of Skh2/J Mice

**Figure 2:**
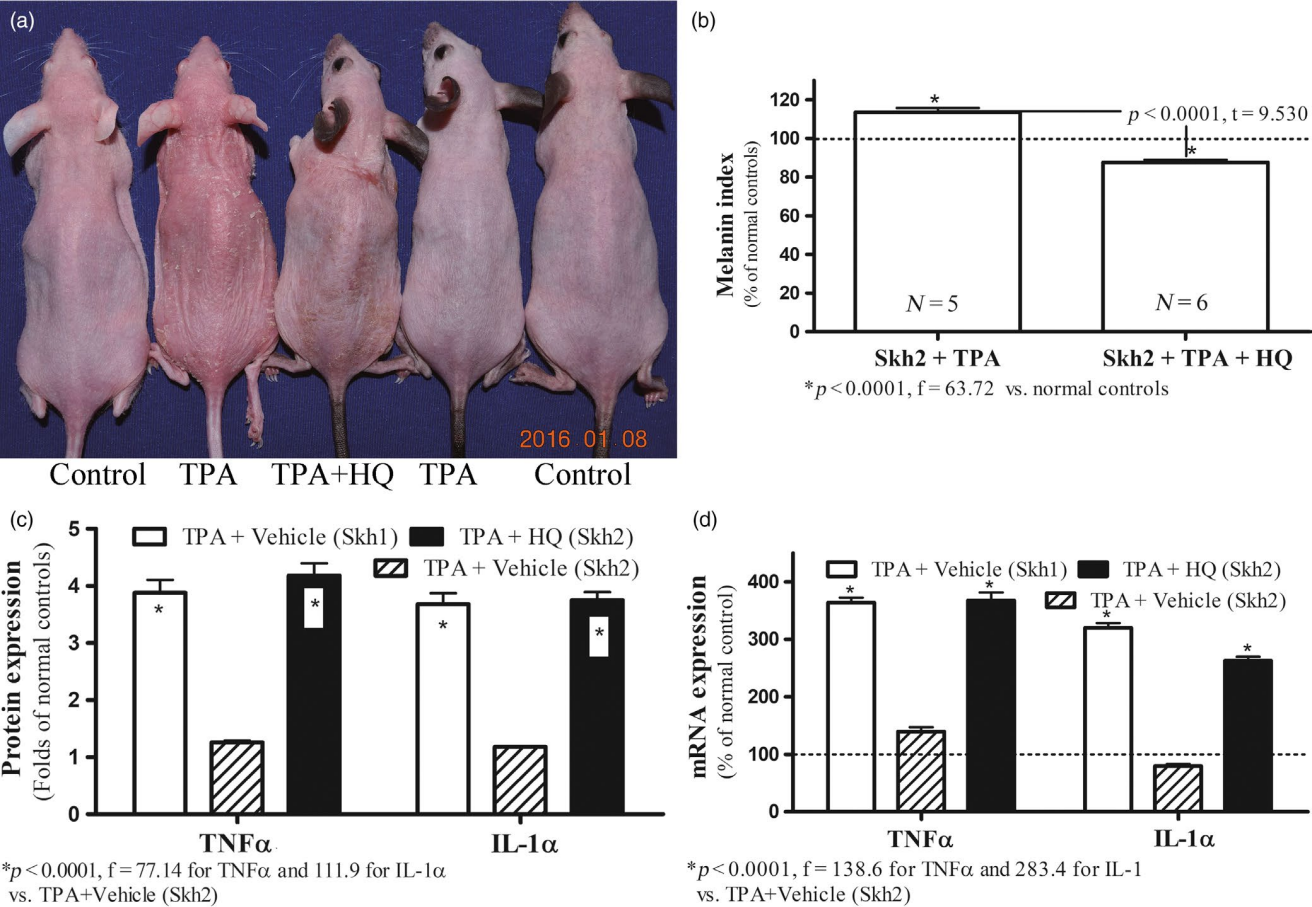
Depigmentation Decreases Inflammatory Thresholds in Pigmented Mice with Irritant Contact Dermatitis (ICD). Figure 2a displays visible changes in inflammation in depigmented Skh2/J in comparison with Skh1 mice. Figure 2b shows epidermal melanin content in TPA‐treated Skh2/J mice, with or without prior depigmentation with topical hydroquinone (HQ). Data are expressed as % of normal Skh2/J mice (setting normal control as 100%, shown in dotted line). Figure 2c compares levels of epidermal TNFα and IL‐1α protein before and after depigmentation with HQ. Expression levels were normalized to respective normal controls, setting normal controls as 1. Data are expressed as fold‐changes from respective normal controls (shown in dotted lines). Figure 2d exhibits changes in epidermal TNFα and IL‐1α mRNA levels after depigmentation. Data are expressed as % of normal controls (shown in dotted lines). One‐way ANOVA with Tukey's multiple comparison test was used to determine the statistical significances among groups. For Figure 2b, unpaired two‐tailed *t* test was also used to determine the significance between the two groups. *p*, *F*, and *t* values are indicated in the figures. *N* = 5 for all groups

**Figure 3:**
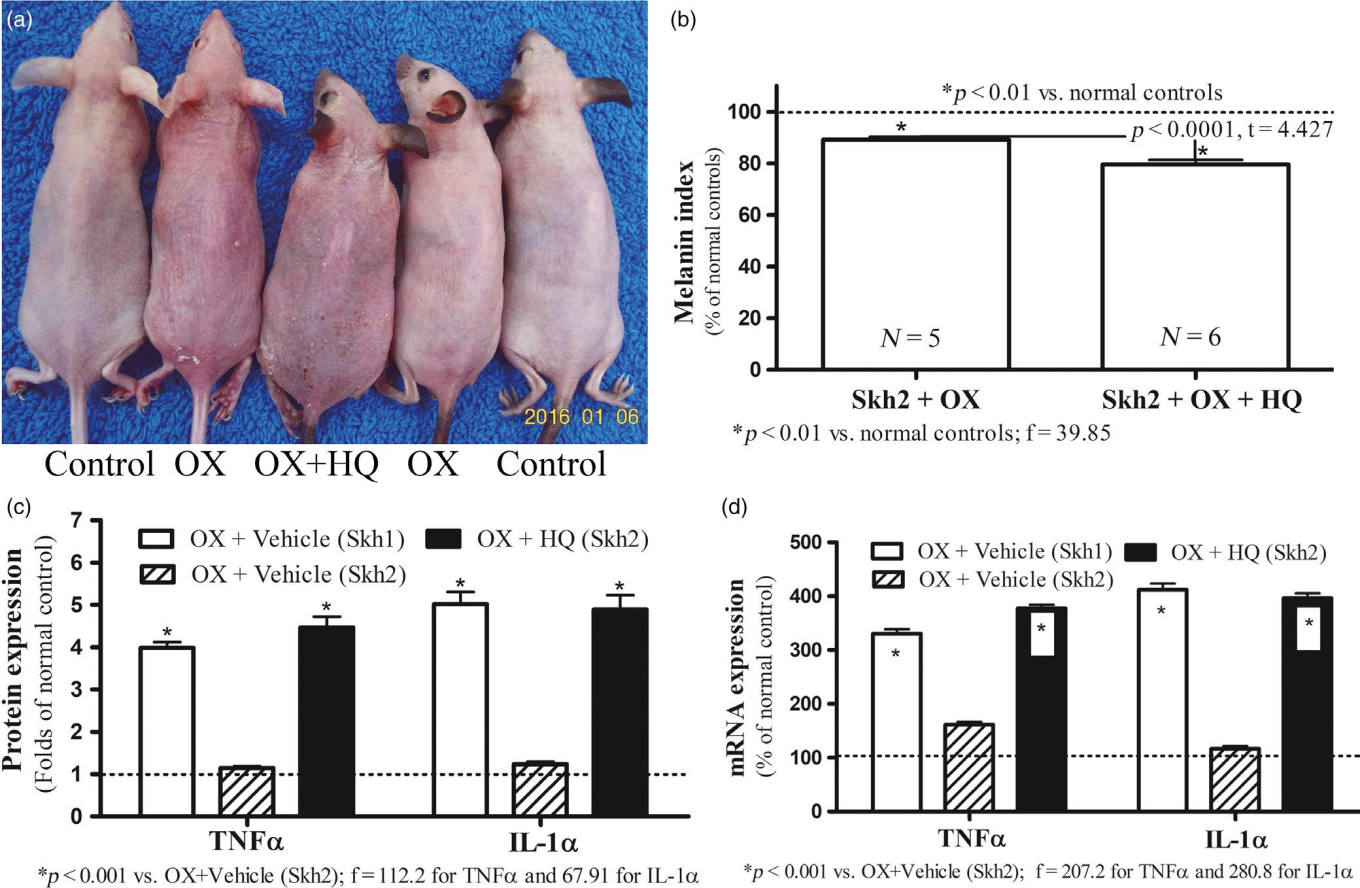
Depigmentation Decreases Inflammatory Thresholds in Pigmented Mice with Atopic Dermatitis (AD). Figure 3a displays macroscopic changes in depigmented Skh2/J mice after hapten (Ox) challenges. Figure 3b shows differences in epidermal melanin content in oxazolone (Ox)‐treated Skh2/J mice, with or without topical applications of hydroquinone (HQ). Data are expressed as % of normal Skh2/J mice (setting normal control as 100%, shown in dotted line). Figure 3c displays changes in epidermal TNFα and IL‐1α protein levels. Expression levels were normalized to respective normal controls, setting normal controls as 1. Data are expressed as fold‐changes from respective normal controls (shown in dotted lines). Figure 3d exhibits changes in epidermal TNFα and IL‐1α mRNA levels. Data are expressed as % of normal controls (shown in dotted lines). One‐way ANOVA with Tukey's multiple comparison test was used to determine the statistical significances among groups. For Figure 3b, unpaired two‐tailed *t* test was also used to determine the significance between the two groups. *p*, F, and t values are indicated in the figures. *N* = 6 for all groups

**Figure 4:**
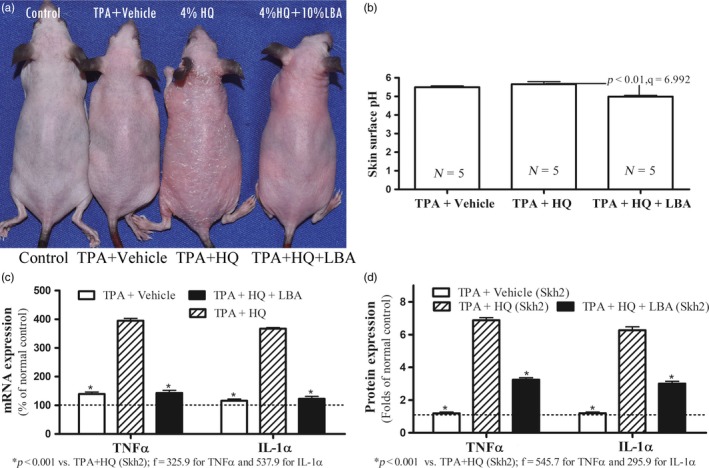
Acidification of Stratum Corneum Increases Inflammatory Thresholds in Depigmented Skh2/J Mice with Irritant Contact Dermatitis (ICD). Figure 4a displays visible changes in inflammation in Skh2/J mice after depigmentation ± acidification with lactobionic acid (LBA). Figure 4b shows differences in skin surface pH after various treatments. Figure 4c displays changes in levels of epidermal TNFα and IL‐1α mRNA levels after depigmentation ± acidification with LBA. Data are expressed as % of control Skh2/J mice, setting controls as 100% shown in dotted line. Figure 4d compares levels of epidermal TNFα and IL‐1α protein after various treatments. Data are expressed as fold‐changes from untreated Skh2/J mice, setting controls as 1 shown in dotted line. One‐way ANOVA with Tukey's multiple comparison test was used to determine the statistical significances among groups. *p*, *F*, and *t* values are indicated in the figures. *N* = 5 for all groups

To determine whether the decreased inflammation in Skh2/J mice could be attributed to the presence of cutaneous pigmentation, we next eliminated melanin on the flanks of Skh2/J mice through repeated, topical applications of hydroquinone (HQ), prior to the induction of either ICD or AD, as above. As previously reported (Man et al., 2014), pigmented, control mice treated with HQ lost all evidence of pigment (see also Figure S1). While topical HQ can occasionally be irritating to human skin, HQ applications to Skh2/J mouse skin provoked neither cutaneous inflammation nor alterations in epidermal function (Figure S2). Macroscopic and quantitative evidence of both TPA‐ and hapten‐induced inflammation became much more apparent in Skh2/J mice that had been previously depigmented, becoming comparable to the levels of inflammation found in similarly treated Skh1 mice (Figures 2a, 3a, 4a). Together, these results demonstrate that the differences in inflammatory thresholds in Skh2/J versus Skh1 mice can be attributed to the presence or absence of cutaneous pigmentation.

### 3.3 Changes in Epidermal Cytokine Levels after Irritant or Hapten Challenges Are Pigment‐Dependent

Challenges with topical irritants or haptens stimulate the production of epidermal pro‐inflammatory cytokines, including TNFα and IL‐1α (Fowler et al., 2003; Funding et al., 2009; Lessard et al., 2013; Sheu et al., 2002), thereby serving as surrogate indicators of cutaneous inflammation. Although there were no differences in the baseline levels of multiple epidermal cytokines in pigmented versus nonpigmented mice (Figure S3), mRNA levels of both epidermal TNFα and IL‐1α increased markedly in Skh1 in comparison with Skh2/J mice after comparable irritant or hapten challenges (Figures 2c,d and 3c,d). Yet, after Skh2/J mice had been previously depigmented with HQ (Figure 2b and 3b) and then challenged with either the irritant or hapten, epidermal cytokine mRNA levels became comparable to those encountered in the epidermis of similarly challenged Skh1 mice (Figure 2c,d and 3c,d). Pertinently and consistent with the lack of evidence of inflammation in HQ‐treated skin, topical applications of HQ alone did not stimulate cytokine production (Figure S2). Together, these results demonstrate that the decreased susceptibility of pigmented murine skin to inflammatory stimuli can be attributed to the presence of epidermal pigmentation.

### 3.4 Decreased Inflammation in Pigmented Mice Can Be Attributed to a Pigment‐dependent Decline in Surface pH

**Figure 5:**
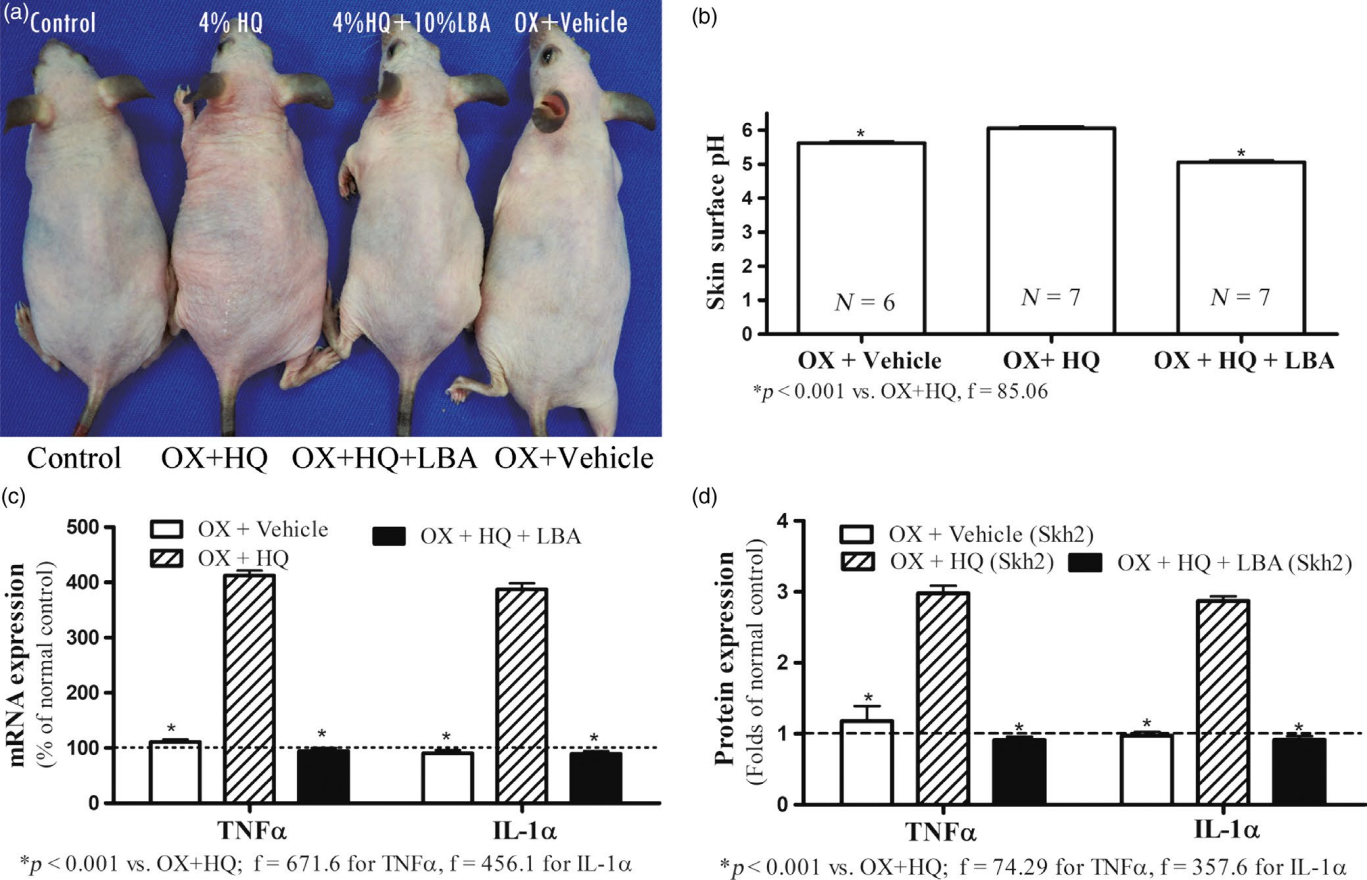
Acidification of Stratum Corneum Increases Inflammatory Thresholds in Depigmented Skh2/J Mice with Atopic Dermatitis (AD). Figure 5a displays visible changes in Skh2/J mice after treatments as in Figure 4, plus repeated hapten (Ox) applications Figure 5b shows differences in skin surface pH. Figure 5c displays differences in epidermal TNFα and IL‐1α mRNA levels. Data are expressed as % of control Skh2/J mice, setting controls as 100% shown in dotted line. Figure 5d exhibits changes in epidermal TNFα and IL‐1α protein levels. Data are expressed as fold‐changes for normal Skh2/J mice, setting controls as 1 shown in dotted line. One‐way ANOVA with Tukey's multiple comparison test was used to determine the statistical significances among groups. *p*, *F*, and *t* values are indicated in the figures. *N* = 7 for all groups, except for Skh2/J + Ox +Veh (*N* = 6)

Our prior studies showed that the lower pH of DP human and pigmented mouse skin accounts for their superior function, because lowering the surface pH of lightly pigmented humans and mice “reset” these functions to levels comparable to pigmented skin (Gunathilake et al., 2009; Man et al., 2014). Hence, we next assessed whether the lower surface pH of DP skin protects against the development of inflammation. Pertinently, while the baseline surface pH of Skh2/J mouse skin was again lower than that of Skh1 mice (Man et al., 2014), the pH of Skh2/J mice increased in parallel with the emergence of inflammation in depigmented animals (Figure 4b and 5b). Though prior HQ‐induced depigmentation of Skh2/J mice again predisposed to greater inflammation and cytokine production after either irritant (Figure 4a,c,d) or hapten challenges (Figure 5a,c,d), topical applications of a nonirritating, acidifying agent, lactobionic acid (LBA) (Hachem et al., 2003, 2010) prevented the subsequent development of inflammation in previously depigmented Skh2/J mouse skin (Figures 4a and 5a). These pH‐dependent changes in inflammatory thresholds were paralleled again by differences in epidermal pro‐inflammatory cytokine production (Figures 4c,d and 5c,d). Similar results were obtained in both the irritant contact dermatitis and atopic dermatitis models of acidified Skh1 mouse skin (Figure S4). Together, these results indicate that the anti‐inflammatory benefits of cutaneous pigmentation can be further attributed to the endowment of pigmented skin with a reduced surface pH.

## 4 DISCUSSION

Shortly after ancestral humans shed their furry mantles and expanded the density of their sweat glands to allow more efficient dissipation of heat, their epidermis became deeply pigmented to protect the skin from UV‐B‐induced toxicity. Yet, whether this increase in cutaneous pigmentation evolved primarily to protect against folic acid degradation (Chaplin & Jablonski, 2009); DNA damage leading to cutaneous malignancies (Brenner & Hearing, 2008); and/or enhanced permeability barrier function (Elias et al., 2009, 2010) is hotly debated. Because our earlier studies had shown that DP humans display superior epidermal barrier function in comparison with their LP counterparts, independent of race, ethnicity, geography, gender, or occupation (Gunathilake et al., 2009; Reed et al., 1995), we have championed the last possibility (Elias & Williams, 2013).

Sub‐Saharan Africa is not only bathed in erythemogenic UV‐B light that is toxic to the skin barrier (Biniek et al., 2012; Elias & Williams, 2013; Haratake et al., 1997), it also was exposed to very low, ambient humidities during the millennia when ancestral humans first became pigmented (Blome et al., 2012; DeMenocal, 1995). Steepening the gradient for transcutaneous water loss in this xeric environment doubtlessly further challenged the skin barrier. Moreover, DP humans, residing at tropical latitudes, also resist the high densities of microbial pathogens that they typically encounter (Mackintosh, 2001), in part due to differences in their skin surface pH (Gunathilake et al., 2009). Notably, microbial pathogens, such as e *S. aureus* and *S. pyrogenes,* proliferate avidly at a higher pH, but poorly at a reduced pH (Elias, 2007; Korting, Hubner, Greiner, Hamm, & Braun‐Falco, 1990). The salubrious benefits of DP for additional epidermal functions can be further attributed to the reduced surface pH of pigmented skin (Elias, 2017), because exogenous acidification of LP humans and nonpigmented mice “resets” these functions (Gunathilake et al., 2009; Man et al., 2014).

Further studies in closely related strains of pigmented (Skh2/J) versus nonpigmented, albino (Skh1), hairless mice shed further light on the mechanisms whereby pigmentation‐induced reductions in skin surface pH are beneficial. As with LP humans, albino mice displayed functional deficits that were comparable to their LP human counterparts; and as in LP humans, lowering the surface pH of albino mice again optimized their cutaneous function (Man et al., 2014). The basis for the superior function of pigmented skin in these mice could be linked to the transfer of melanin granules of different sizes from melanocytes to keratinocytes. These granules are much larger in DP humans and mice than are the “crumbly” granules that are delivered to keratinocytes in LP human and mouse skin (Jimbow, Quevedo, Fitzpatrick, & Szabo, 1976; Szabo, Gerald, Pathak, & Fitzpatrick, 1969). Once inside keratinocytes, all melanin granules, regardless of size, are captured within acidic phagolysosomes (Ando et al., 2012; Boissy, 2003). Then, whereas the smaller, “crumbly” granules of LP humans and animals are rapidly degraded, the larger granules of DP humans and pigmented mice instead persist high into the epidermis (Jimbow et al., 1976), where encircling phagolysosomes eventually release their cargo of melanin granules and protons (Man et al., 2014).

Hence, we proposed that the aggregate functional stressors that were prevalent in Africa during the evolution of *Homo sapiens* could have further selected for the initial evolution and subsequent persistence of dense epidermal pigmentation in ancestral humans (Elias & Williams, 2013, 2016). Despite the substantial metabolic costs of pigment production, this trait has persisted, likely because of the multiple benefits of dark pigmentation for cutaneous function (see below).

In these studies, we explored the merits of clinical studies, which have suggested that DP humans display both a greater propensity and a reduced threshold to develop inflammatory dermatoses. We showed first that the extent of inflammation is regulated by the presence or absence of pigmentation; and further, that the extent of inflammation is linked to the role of dark pigmentation in reducing the pH of the outer epidermis (Gunathilake et al., 2009; Man et al., 2014). These observations illuminate the key role of surface pH as a master regulator of permeability barrier homeostasis, stratum corneum integrity, and antimicrobial defense, a suite of benefits to which cutaneous inflammation now can be added. Although not yet assessed, it remains possible that changes in less metabolically expensive acidifying mechanisms; for example, the sodium proton transporter, NHE1, and/or one or more secretory phospholipases could contribute to strain‐specific differences in surface acidification. In further mechanistic studies, we have shown that major benefits of a low surface pH are (a) deactivation of pro‐inflammatory serine proteases (kallikreins), which damage epidermal structure and function (Elias, 2017; Jang et al., 2016; Voegeli et al., 2009); and 2) while simultaneous activation of two, beneficial ceramide‐generating enzymes (Uchida et al., 2000).

Our results must be tempered by two facts: (a) mice are distant relatives of humans, although mutant and genetically engineered mouse models have provided a remarkable array of insights about organ function in normal humans; and (b) although it is indisputable that innate and adaptive immune components of the inflammatory response are operative in both species, their details could differ. Nonetheless, we deployed these murine models to gain insights into the role of pigmentation in epidermal function. Despite the fact that the extent of inflammation can be difficult to assess in darkly pigmented skin, our results were unambiguous. Skh2/J mice display a broad dispersion of pigmentation across their rather pale‐appearing truncal skin (Man et al., 2014), allowing ready comparisons of the extent of inflammation. By multiple macroscopic, functional, and molecular parameters, Skh2/J skin displayed a markedly reduced propensity to develop inflammation in comparison with their pale‐skinned (Skh1) counterparts. Indeed, our observations of differences in visible inflammation were inevitably paralleled by changes in dermal inflammation and levels of pro‐inflammatory cytokines.

The broad suite of functional benefits that are attributable to DP call into question the widely held belief that DP humans are more susceptible to inflammatory dermatoses (Abuabara et al., 2019; DeFelice & Yousef, 2008; Moore et al., 2004; Vachiramon et al., 2012; Williams et al., 1995). These studies raise the possibility that such reported differences in prevalence and severity could instead reflect sociocultural or environmental conditions, including increased exposure to pathogens and allergens (Wegienka et al., 2012; Williams et al., 1995). Moreover, such crowded living conditions, often amplified by surrounding, tall structures that block sunlight, reduce exposure to the salubrious benefits of suberythemogenic UV‐B (Thyssen, Zirwas, & Elias, 2015). Though the adverse impact of pigmentation in the above‐cited clinical studies likely reflects one or more of these factors, additional studies are needed to assess whether such sociocultural and/or environmental disadvantages can account for the clinical disparities observed in prior epidemiologic studies.

In summary, while studies in mice may not always reflect similar propensities in humans, our results suggest that resistance to inflammation can now be added to the growing list of ways in which dark pigmentation likely evolved to support cutaneous function in ancestral humans.

## CONFLICT OF INTEREST

The authors have no conflicts of interest to declare.

## Data Availability

No datasets were generated or analyzed during the current study.
