# Supplementary material for: By protecting against cutaneous inflammation, epidermal pigmentation provided an additional advantage for ancestral humans
Source: Evol Appl. 2019 Sep 24;12(10):1960–70. doi: 10.1111/eva.12858 (PMC6824065; doi:10.1111/eva.12858)

Supplemental Figure 1. Topical hydroquinone decreases epidermal melanin pigment in Skh2/J mice. Skh2/J mice were treated topically with either 4% hydroquinone (HQ) or vehicle (ethanol) alone twice daily for 10 days. Eighteen hours after last HQ application, skin samples were taken for Fontana-Masson staining.

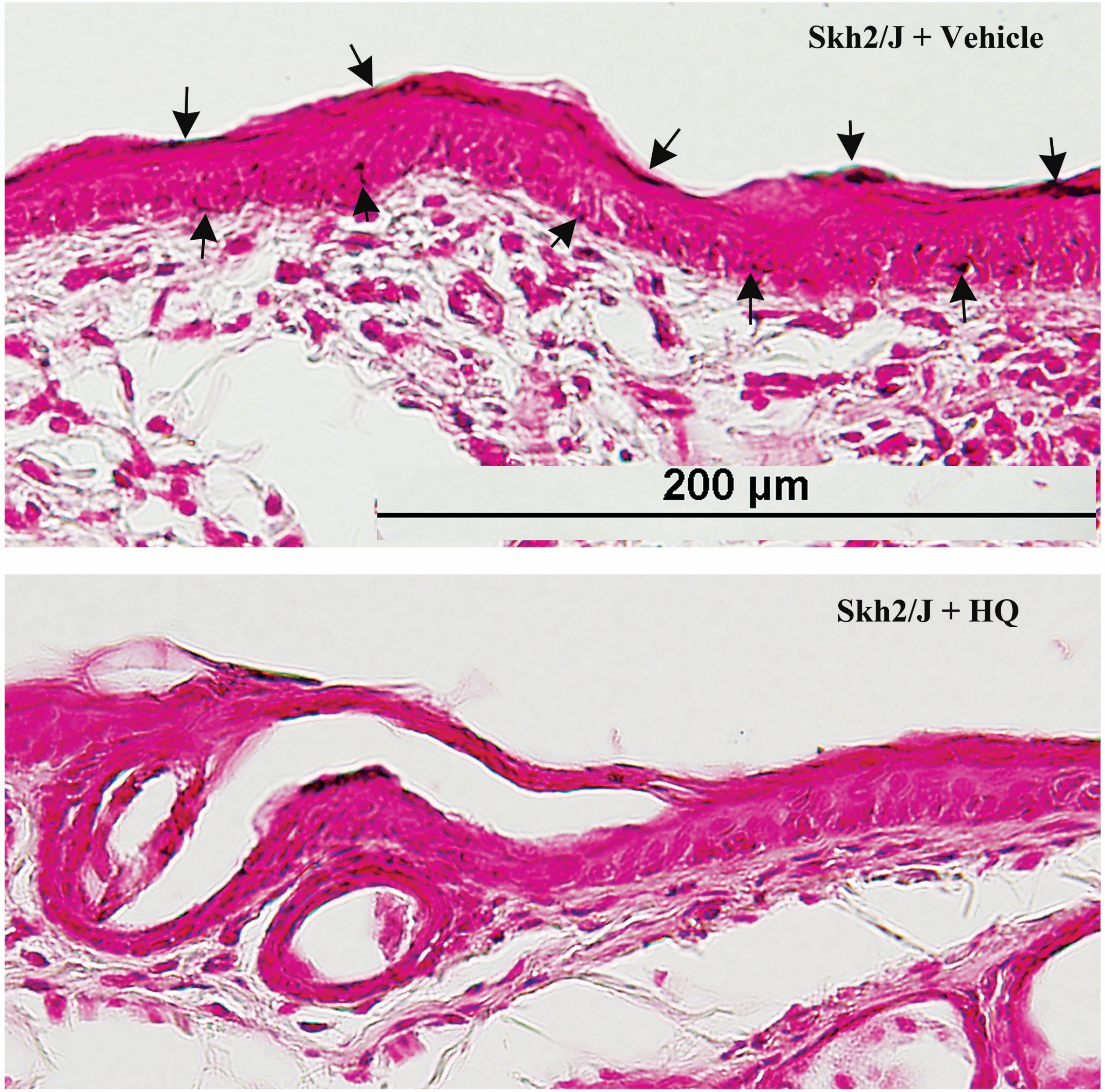

Supplemental Figure 2. Topical applications of hydroquinone alone alter neither epidermal function nor levels of inflammatory cytokines. Skh2 mice were treated topically with either 4% hydroquinone (HQ) or vehicle (ethanol) alone twice daily for 10 days. Eighteen hours after last HQ application, epidermal function was measured and skin samples were taken for assessing expression levels of inflammatory cytokines. Suppl. Fig. 2a shows TEWL rates and stratum corneum hydration levels in vehicle- versus HQ-treated mice. Suppl. Fig. 2b and c demonstrate expression levels of epidermal mRNA and protein of cytokines, respectively. Numbers of mice are indicated in the figures.

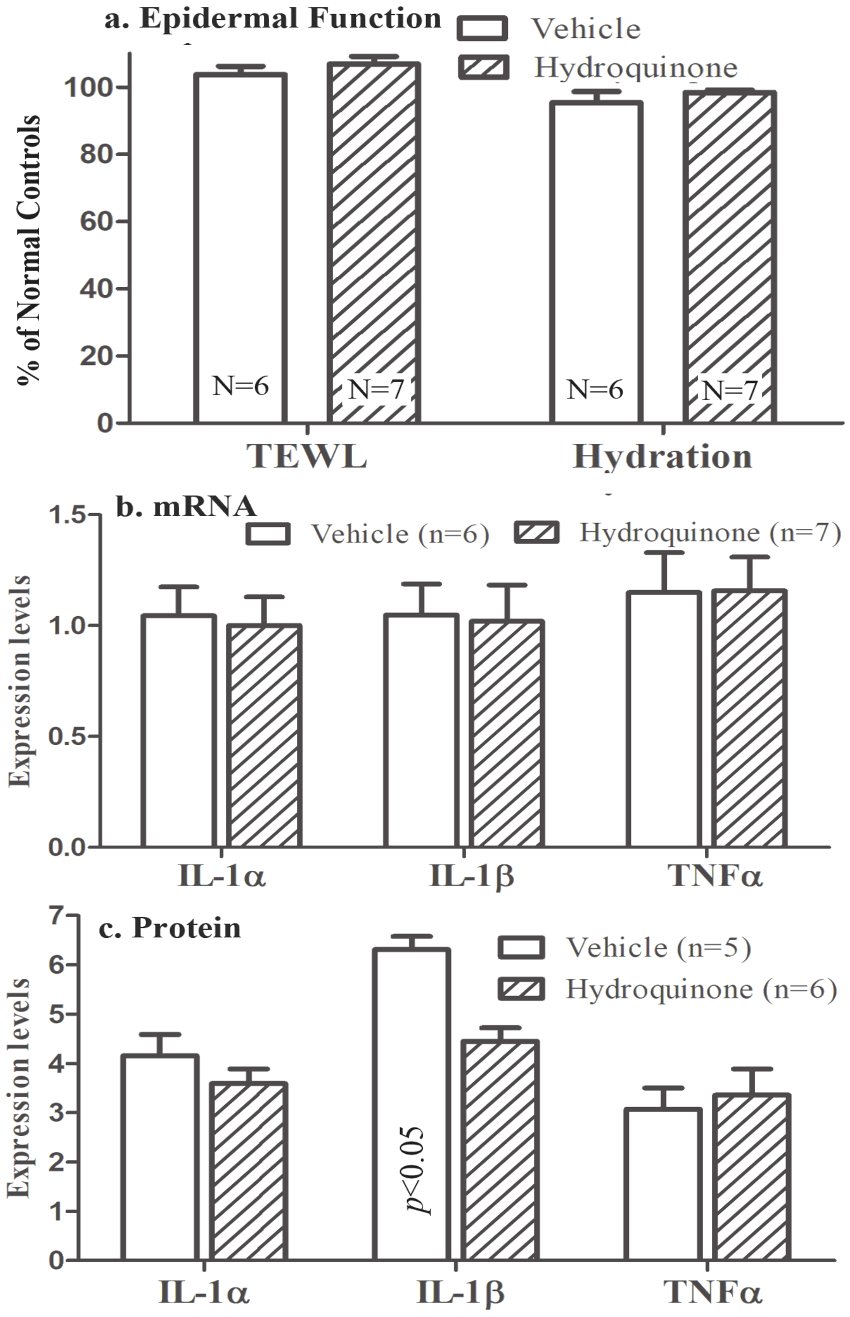

Supplemental Figure 3. Baseline levels of inflammatory cytokines are comparable in Skh1 and Skh2/J mouse epidermis. Suppl. Fig. 3a depicts protein levels of epidermal cytokines in Skh1 versus Skh2 mice while 3b displays mRNA levels of epidermal cytokines in Skh1 versus Skh2 mice. Numbers of mice are indicated in the figures.

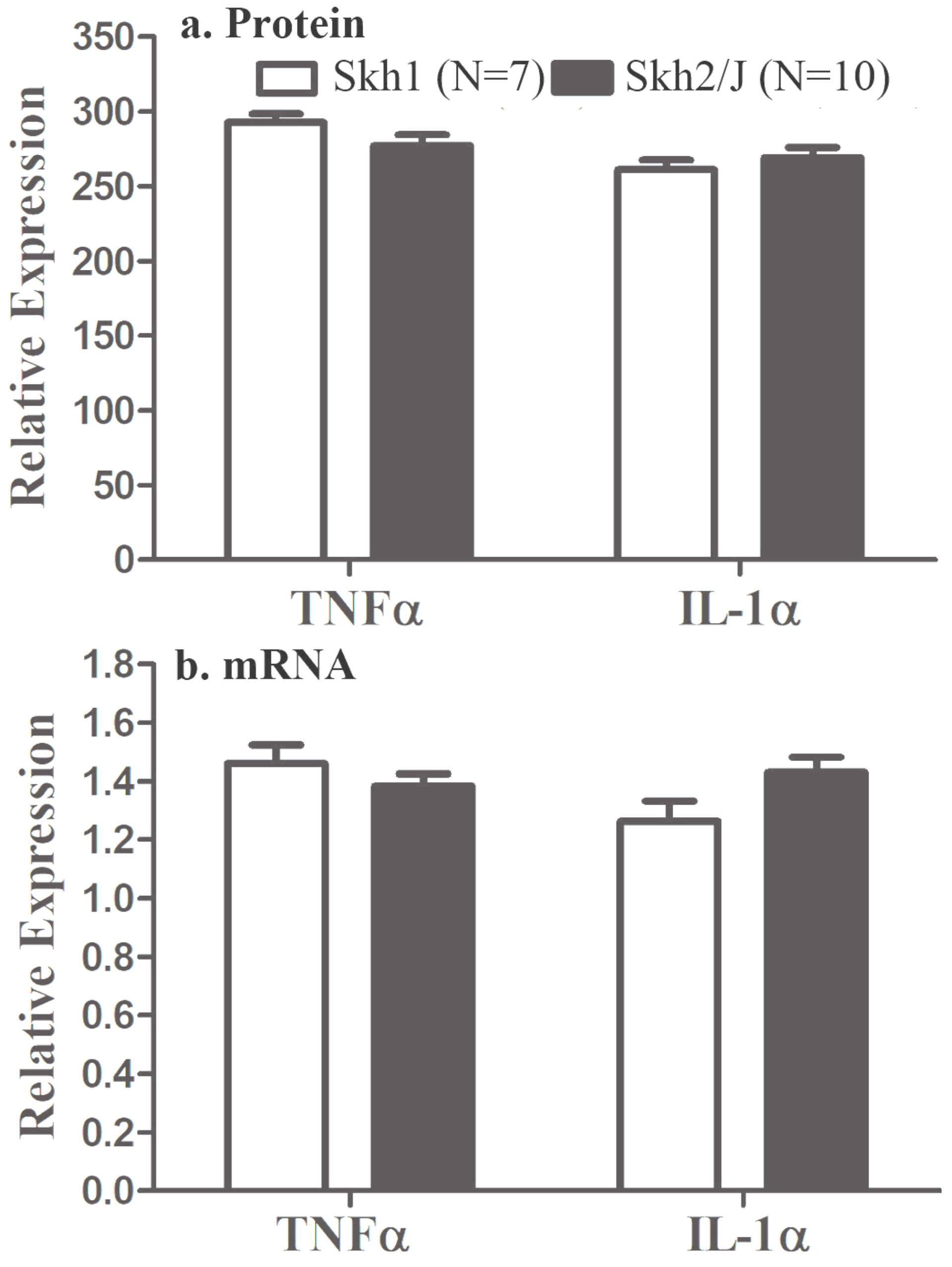

Supplemental Figure 4. Acidification of Stratum Corneum Increases Inflammatory Thresholds in Skh1 Mice.
Expression levels of mRNA for TNFα and IL-1α were measured in both irritant contact dermatitis and atopic dermatitis models of Skh1 mice with or without acidification of the stratum corneum. Data are expressed as fold increase over normal controls of Skh1 mice. One-way ANOVA with Tukey's Multiple Comparison Test was used to determine the statistical significances among groups. In OX model, F= 109.7 for TNFα, and F=139.5 for IL-1α; In TPA model, F=160.1 for TNFα, and F=69.88 for IL-1α. *P* values are for TPA or OX with vs. without acidification. N=6 for all groups.

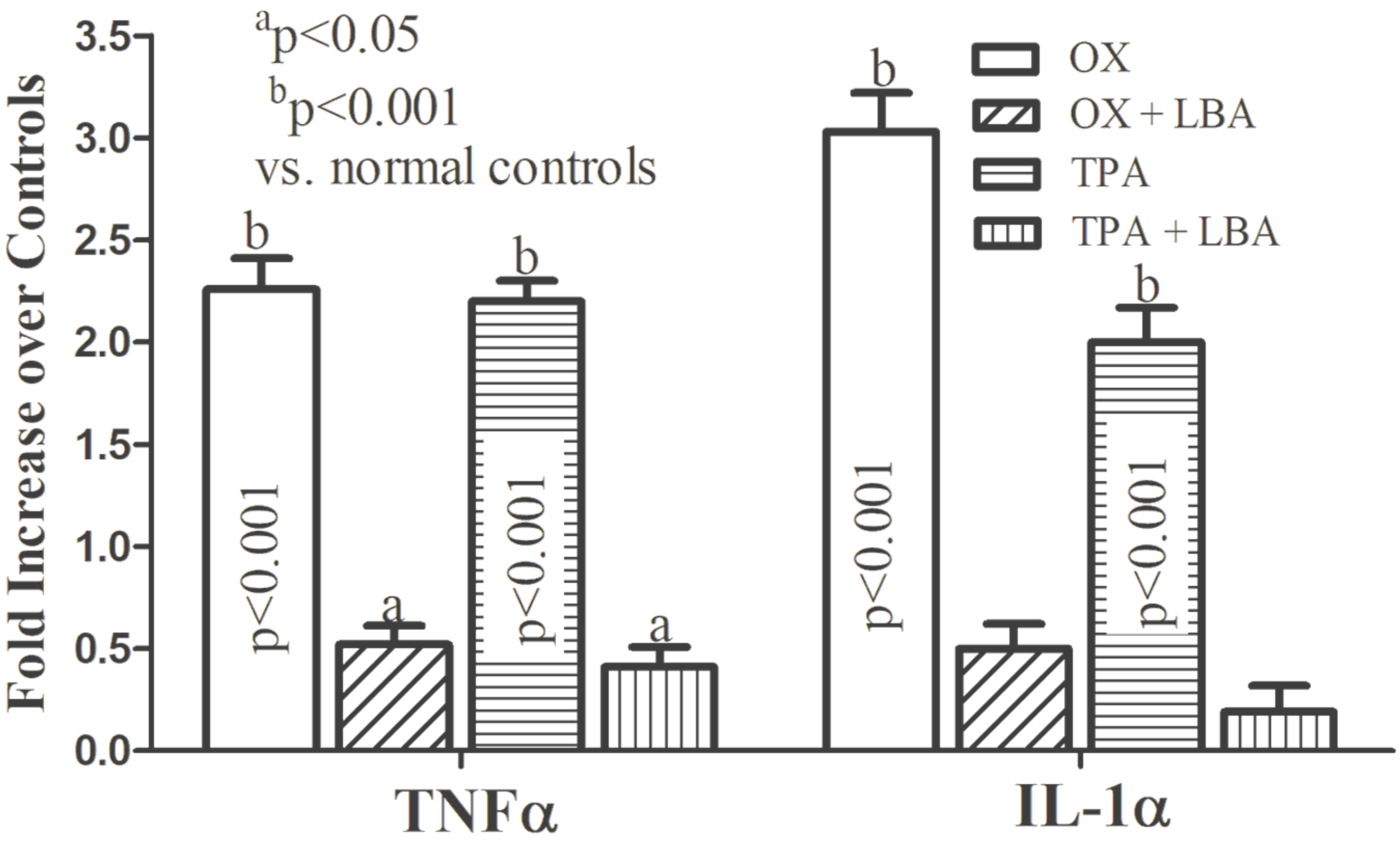

Supplement: Supplementary file 1 [file EVA-12-1960-s001.pdf]
